# Effects of Enriched-in-Oleuropein Olive Leaf Extract Dietary Supplementation on Egg Quality and Antioxidant Parameters in Laying Hens

**DOI:** 10.3390/foods12224119

**Published:** 2023-11-13

**Authors:** Georgios A. Papadopoulos, Styliani Lioliopoulou, Nikolaos Nenadis, Ioannis Panitsidis, Ioanna Pyrka, Aggeliki G. Kalogeropoulou, George K. Symeon, Alexios-Leandros Skaltsounis, Panagiotis Stathopoulos, Ioanna Stylianaki, Dimitrios Galamatis, Anatoli Petridou, Georgios Arsenos, Ilias Giannenas

**Affiliations:** 1 Laboratory of Animal Husbandry, Faculty of Veterinary Medicine, Aristotle University of Thessaloniki, 54124 Thessaloniki, Greece; slioliopo@vet.auth.gr (S.L.); arsenosg@vet.auth.gr (G.A.); 2 Laboratory of Food Chemistry and Technology, School of Chemistry, Aristotle University of Thessaloniki, 54124 Thessaloniki, Greece; niknen@chem.auth.gr (N.N.); ioannapyrka@chem.auth.gr (I.P.); aggelkal@chem.auth.gr (A.G.K.); 3 Laboratory of Nutrition, Faculty of Veterinary Medicine, Aristotle University of Thessaloniki, 54124 Thessaloniki, Greece; panitsid@vet.auth.gr (I.P.); igiannenas@vet.auth.gr (I.G.); 4 Institute of Animal Science, Hellenic Agricultural Organisation-DEMETER, 58100 Giannitsa, Greece; gsymeon@elgo.gr; 5 Department of Pharmacognosy and Natural Products Chemistry, Faculty of Pharmacy, University of Athens, 15771 Athens, Greece; skaltsounis@pharm.uoa.gr (A.-L.S.); stathopan@pharm.uoa.gr (P.S.); 6 Laboratory of Pathology, Faculty of Veterinary Medicine, Aristotle University of Thessaloniki, 54124 Thessaloniki, Greece; stylioan@vet.auth.gr; 7 Department of Animal Science, School of Agricultural Sciences, University of Thessaly, 41500 Larissa, Greece; dgalamatis@uth.gr; 8 Laboratory of Evaluation of Human Biological Performance, School of Physical Education and Sport Science at Thessaloniki, Aristotle University of Thessaloniki, 54124 Thessaloniki, Greece; apet@phed.auth.gr

**Keywords:** olive, olive byproduct, olive leaf extract, poultry, laying hens, antioxidants, egg quality

## Abstract

The objective of the present study was to evaluate the effects of an olive leaf extract obtained with an up-to-date laboratory method, when supplemented at different levels in laying hens’ diets, on egg quality, egg yolk antioxidant parameters, fatty acid content, and liver pathology characteristics. Thus, 96 laying hens of the ISA-Brown breed were allocated to 48 experimental cages with two hens in each cage, resulting in 12 replicates per treatment. Treatments were: T1 (Control: basal diet); T2 (1% olive leaf extract); T3 (2.5% olive leaf extract); T4 (Positive control: 0.1% encapsulated oregano oil). Eggshell weight and thickness were improved in all treatments compared to the control, with T2 being significantly higher till the end of the experiment (*p* < 0.001). Egg yolk MDA content was lower for the T2 and T4 groups, while total phenol content and Haugh units were greater in the T2. The most improved fatty acid profile was the one of T3 yolks. The α-tocopherol yolk content was higher in all groups compared to T1. No effect was observed on cholesterol content at any treatment. Based on the findings, it can be inferred that the inclusion of olive leaf extract at a concentration of 1% in the diet leads to enhancements in specific egg quality attributes, accompanied by an augmentation of the antioxidant capacity.

## 1. Introduction

The prohibition imposed by the European Union on the utilization of antibiotic growth promoters, coupled with the increased consciousness among the public regarding the quality standards associated with chicken products, has spurred manufacturers to investigate natural feed additives as potential substitutes for antibiotics [1]. It is generally acknowledged that eggs are the primary animal protein source for humans and are highly nutritious, exhibit a multitude of valuable biochemical properties, and are known worldwide for their substantial antioxidant capabilities [2]. Waste materials and byproducts are used to extract beneficial nutrients to meet customer and societal needs for high-quality, safe, and environmentally friendly processed foods [3,4,5]. Laying hens may also be exposed to stimuli that increase oxidative stress at different phases of production. Oxidative stress affects laying hens’ health and productivity [6,7]. Towards this direction, studies using antioxidant supplements were able to reverse the color of the shell caused by oxidative stress [6,8,9]. Thus, various naturally occurring phytochemicals provided through feed have received attention as poultry antioxidants in recent years [10].

It is known that spices and herbs exhibit the most substantial concentration of polyphenol chemicals when measured by weight [11]. Polyphenols can impede oxidation by preventing free radical production. They may also inhibit oxidation by scavenging free radicals [12]. Furthermore, they improve the antioxidant status of animals by raising the levels of vitamins and antioxidant enzymes in the blood and muscles [13,14]. Several beneficial effects in laying hens have been observed. Specifically, supplementing layer chickens’ diets with tea polyphenols enhanced their productivity, the quality of their eggs, and their ability to withstand induced oxidative challenge [9]. Administering tea polyphenols to laying hens enhanced egg production and egg albumen quality [15]. On the other hand, elevated levels of tea polyphenols had a negative impact on the quality of both eggshell and albumen [16]. Elsewhere, green tea polyphenols improved the shape and antioxidant capacity of the uterine in layers subjected to induced oxidative stress, thereby improving eggshell color [9,17]. Others have demonstrated that laying hens’ performance, egg quality, and intestine morphometric characteristics were all enhanced by oregano essential oil dietary supplementation [18].

Polyphenol-rich plant leaves have also been studied. The rate and quality of eggs increased with the addition of eucalyptus leaves to the diet. It also boosted the hens’ health and blood antioxidant levels. Eucalyptus leaves in the diet protected chickens’ liver cells against ethanol-induced oxidative damage [19]. The use of powdered eucalyptus leaves increased the number, mass, feed conversion ratio, and breaking strength of eggs while lowering the laying chickens’ heterophil/lymphocyte ratio [20]. More recently, in a separate study, the utilization of Mulberry leaf extract resulted in a reduction of yolk triglyceride and total cholesterol contents while concurrently enhancing both egg yolk color and eggshell strength [21]. Olive leaves possess a substantial antioxidant potential due to their abundance of secoiridoids, simple phenols, phenylethanoids, hydroxycinnamic acid derivatives, and flavonoids [22]. In olive varieties, oleuropein is typically the most abundant phenol. As a component of the phenolic segment of olive leaves, oleuropein is readily isolated [23]. Ahmed et al. (2018) [24] showed that egg yolk color was higher when the hens were fed the greatest level of leaf extract (150 mg/kg) supplementation. Egg yolk cholesterol and saturated fatty acid concentrations were lower for those hens supplemented with the extract. On the other hand, n-3 and n-6 fatty acids were higher while increasing levels of the extract fed [24]. The olive leaf or the use of its extract had no effect on laying hen performance, according to Rezar et al. [25]. In comparison to those fed unsupplemented diets, laying hens fed diets with olive leaf powder had higher body weights and darker egg yolks [26]. Elsewhere, olive leaf powder added to the diet of Japanese quails increased egg production while decreasing metabolic parameters such as serum lipids and cholesterol [27]. Recently, our research group reported that the supplementation of olive leaf extract at the level of 1% in broilers alleviated oxidation in broiler meat [5]. Therefore, it can be deduced that any oxidative challenge experienced by laying hens will have an impact on both their metabolism and the parameters associated with egg quality. This aspect holds significance from a welfare perspective, as contemporary consumers demonstrate concern not only for the quality of obtained products but also for the well-being and health of animals. It should be noted that the results of earlier studies are inconsistent, and only a few have followed a conclusive approach, investigating the analytical composition and polyphenol content of the olive leaf extract and the respective effect in the feed and the eggs produced. Overall, it can be hypothesized that olive leaf extract supplementation could be beneficial for laying hens’ health and egg quality characteristics. In view of this, we have implemented in the current study the use of an olive leaf extract that was obtained with an up-to-date laboratory method to ensure an adequate concentration of active phenolic compounds. Moreover, to investigate whether egg quality characteristics could be negatively affected by a pro-oxidant effect of polyphenols, the tested olive leaf extract was evaluated at two levels of supplementation. An additional experimental group, supplemented with oregano essential oil, was used as a positive control group based on the documented effects on laying hens’ performance.

## 2. Materials and Methods

### 2.1. Ethical Considerations

The experimental procedures were approved by the Research Committee of Aristotle University of Thessaloniki, Greece (approval number 246648/15-10-2021; project number 72623). The experiment’s animal phase was designed with careful consideration of all welfare factors specified in the Good Farming Practice Guidelines, according to Directive 2010/63/EC and Commission recommendation 2007/526/EC.

### 2.2. Raw Materials, Animals, Diets and Experimental Design

The extraction and the HPLC–DAD analyses were carried out at the Department of Pharmacognosy and Natural Products Chemistry, Faculty of Pharmacy, University of Athens, Greece. The extraction of olive leaves was performed according to our previous work [5], and the extract obtained was condensed and dried prior to HPLC-DAD analyses, following COI/T.20/Doc. No 29/Rev.1 2017 elution protocol [28]. The extract contained the active ingredients oleuropein (22.84 g/100 g extract), luteolin-7-O-glucoside, hydroxytyrosol, and verbascoside (<0.8 g/100 g extract), and also triterpenic acids (maslinic and oleanolic acid, 4.97 and 1.08 g/100 g extract, respectively).

The oregano oil used in the present study was prepared by the vis-Naturalis company. According to the latter, it contained 78–85% carvacrol. It was derived after microencapsulation [29] following a spray drying technique, which provided a white powder as the end product.

The investigation was conducted in Galatista, a municipality in Chalkidiki, Greece, in a certified poultry house. The experimental enclosures utilized for accommodating the laying hens were of dimensions 41 cm by 41 cm, resulting in an area of 840.5 cm^2^ per hen. This allocation of space exceeded the minimum requirement mandated by the European Union legislation (750 cm^2^ available space per hen). In total, 96 laying hens of the ISA-Brown breed, aged 45 weeks, were allocated to 48 experimental cages with two hens in each cage. Each treatment consisted of 12 replicate cages. Diets were formulated to meet nutrient specifications provided by the specified laying hen breeder’s guidelines [30]. The average temperature during the experimental period was maintained between 18–22 °C, and relative humidity was registered between 55–70%. A 14 to 16 h of light duration was applied based on the specified laying hen breeder’s guidelines [30]. The experimental duration was 6 weeks, preceded by a one-week adaptation period. The dietary treatments included T1-control, no supplement test product; T2-1% olive leaf extract supplementation; T3-2.5% olive leaf extract supplementation; T4-0.1% oregano oil supplementation. All diets were prepared by Vis-Naturalis, Gennimata 17 str-Kalamaria, Thessaloniki, 55132, Greece. The main ingredients and calculated nutrient analysis of the diets are shown in Table 1.

### 2.3. Feed Color, a_w_, and Proximate Composition Analyses

Feed samples of 400 g were obtained from the main batch (1 tn) and separated into three homogeneous parts. Each sub-sample obtained was analyzed for color (MiniScan XE Plus D/8S Color Analyzer Colorimeter Spectrophotometer, Hunterlab, VA, USA), and ∆E values were evaluated [31]. Regarding a_w_, it was measured thrice at 25 °C using an Aqualab 3TE water activity meter (Decagon Devices Inc., Pullman, WA, USA). Measurements of moisture, crude fat, crude protein, and crude ash were based on a previous method [32].

### 2.4. Feed Fatty Acid Profile Analysis

The fatty-acid content of feed samples was analyzed with gas chromatography, according to Vasilopoulos et al. [33].

### 2.5. Feed Antioxidant Parameters

Initially, each feed sub-sample was extracted once. The extracts obtained were further processed. The antioxidant parameters measured were the following: Total phenol content (TPC), Total flavonoid content (TFC), DPPH^●^ scavenging, and Cupric ion Reducing Antioxidant Capacity (CUPRAC), as in previously published methodology [5,34].

### 2.6. Feed Content in Oleuropein

The methanolic extracts obtained were analyzed chromatographically using a Shimadzu Nexera X2 UHPLC System (Shimadzu Corporation, Kyoto, Japan). More details about the UHPLC system are described in our previous published work [5]. For UHPLC data acquisition and analysis, the exact methods are further described in Tsimidou et al. (2019) [35]. Quantification of oleuropein was carried out via the construction of a calibration curve (y = 617.68x + 4947.3, R^2^ = 0.9989) using a set of standard solutions (4.55–910 mg/L) analyzed at 280 nm.

### 2.7. Characterization of Essential Oil Content and Composition

Hydrodistillation was used for oil extraction and disruption of the wall material. The procedures were according to a previous study [36].

### 2.8. Olive Leaf Extract Characterization

For ɑ_w_, moisture content, TPC, TFL, and antioxidant capacity of olive leaf extract, the methods followed were the same as described previously for feed extracts. The content of individual constituents was characterized chromatographically (see Section 2.3).

### 2.9. Egg Quality Parameters

The following egg quality parameters were measured: egg weight (g), yolk weight (g), albumen weight (g), eggshell weight (g), eggshell thickness (mm), longitudinal and transverse axes (mm), shape index, eggshell color, yolk color and Haugh units. Yolk color was measured both with the DSM YolkFanTM scale and by a Chroma meter CR-410 (Konica Minolta, Osaka, Japan), which was used for L*, a*, and b* color values evaluation.

### 2.10. Yolk Lipid Oxidation

It was assessed by TBARS assay (Thiobarbituric Acid Reactive Substances). Yolk samples weighing 1 g were homogenized with 8 mL of 5% Trichloroacetic acid (TCA) and 5 mL of 0.8% Butylated hydroxytoluene (BHT) solution in hexane. The homogenates were centrifuged (3000 rpm, 3 min), and following centrifugation, 2.5 mL of the bottom layer were collected. The 2.5 mL aliquots collected were transferred in tubes, and 1.5 mL of 0.8% Thiobarbituric Acid aqueous solution was added to each tube. Next, the tubes were incubated in a water bath (70 °C, 30 min) and cooled immediately after the incubation. Absorbance was measured in a spectrophotometer (UV-1700 PharmaSpec, Shimadzu, Japan) at 532 nm. Results were measured as ng of Malondialdehyde (MDA) per gram of yolk (ng MDA/g yolk).

### 2.11. Yolk Total Phenol Content

The total phenol content of yolks was measured by the Folin–Ciocalteu assay, according to a protocol by Shang et al. [37]. Results were presented as μg of Gallic Acid equivalent (GAE) per g of dried yolk.

### 2.12. Yolk Total Antioxidant Capacity (TAC) (Phosphomolybdate Method)

Yolk extracts were prepared with the same method described for Total Phenol Content. The phosphomolybdate method was applied to assess TAC, following the protocol by Prieto et al. [38]. Results were expressed as TAC (%).

### 2.13. Yolk Fatty Acid Profile Analysis

Gas chromatography was used for fatty acid profile analysis of egg yolk. Before extraction, 10 μL of 200 mg/mL pentadecanoic acid (Sigma, St. Louis, MO, USA) in chloroform as internal standard were mixed with 50 mg of yolk. Extraction was performed according to Folch et al. [39].

### 2.14. Yolk Cholesterol and α-Tocopherol Content

Determination of cholesterol (CHO) and α-Tocopherol (α-T) content in egg yolks was performed in accordance with the protocol of Botsoglou et al. (1998) [40]. Normal phase HPLC-DAD-FLD was performed for the simultaneous determination of CHO and α-Τ in egg yolk samples. The system used for Chromatographic analysis was a SpectraSystem SCM1000 HPLC system, equipped with a SpectraSYSTEM P4000 pump, a MIDAS autosampler from Spark (Waanderveld, Holland), a communication unit SpectraSYSTEM SN4000, a UV-visible diode array Thermo Separation Products UV6000 detector (Thermo Fischer Scientific, Waltham, MA, USA) coupled to an FL2000/FL3000 FASMA 502 fluorescence detector supplied by Rigas Labs (Thessaloniki, Greece). Data analysis was performed using the ChromQuest ver. 5.0 software (ThermoFischer Scientific, Waltham, MA, USA). The separation was carried out on a normal phase, 250 × 4 mm i.d., 5 μm, LiChrospher-SI column (MZ Analyzentechnik, Mainz, Germany). The elution system consisted of a mixture of hexane:2-propanol (99:1 *v*/*v*), with the following elusion conditions: isocratic elution; flow rate of 1.1 mL/min; injection volume: 20 µL. Standard solutions of CHO (100–1000 µg/mL) and α-T (1–10μg/mL) were prepared in hexane. Prior to injection, they were filtrated through PTFE hydrophobic filters (25 mm, 0.45 um) prior to injection. Samples were saponified twice and then analyzed in duplicate (CV% ≤ 2, *n* = 2 × 2). Quantification was carried out using a CHO calibration curve at 210 nm (y = 316.99x + 2E + 06, R2 = 0.9907) and α-T calibration curve and fluorescence detection (λex 294 nm; λem-1 330 nm) (y = 1328.8x + 6487.4, R2 = 0.9905).

### 2.15. Pathology Evaluation

Randomly selected laying hens (*n* = 6 per treatment) were euthanized and subjected to a complete necropsy within 30 min after slaughter. The condition of the carcasses displayed minimal to no signs of autolysis. The liver and genital tract were grossly examined. Livers were examined for enlargement, discoloration, and variations in texture consistency. During the necropsy, liver samples were collected, fixed in 10% neutral buffered formalin, and stained with hematoxylin and eosin (HE). The liver sections were screened for six known microscopic alterations, including hepatocellular vacuolization, hepatocellular necrosis, vascular lesions, inflammation, and biliary reaction. Each histologic modification was scored with a categorical scoring from 0 to 3. Within the scoring system, a score of 0 corresponded to no detection of the specific lesion, while a score from 1–3 denoted greater levels of tissue damage and/or the extent of the observed changes.

### 2.16. Statistical Analysis

Statistical analysis of data was performed with the use of SPSS software (SPSS 25.0 Version, Chicago, IL, USA). Statistical difference was set at *p* < 0.05, and results were presented as average values ± standard deviation (SD). For analyzing the effects of treatments on the tested variables, one-way ANOVA was employed. Post hoc evaluation was conducted with Tukey’s test. The effect of the treatments on hepatic vacuolization scoring in laying hens was evaluated with Chi-square analysis.

## 3. Results

### 3.1. Feed Color, a_w_, and Proximate Composition

The color values L*, a*, b*, water activity (a_w_), and proximate composition of feed samples from the treatments are presented in Table 2.

Calculation of the ∆E values for the three different feeds compared to T1 indicated that the addition of the olive leaf extract affected the appearance of the feeds due to its yellow–green coloration and no influence was found for the addition of the encapsulated oregano oil. In agreement with previous findings for preparing broilers’ feeds [5], the corresponding ∆E values indicated that mixing was adequate and that T3 contained a higher dose of the extract than T2. Although higher than those obtained in our previous work, water activity and moisture content values were still relatively low and in agreement with published values [41]. Fat, protein, and ash content were rather similar in all treatments.

### 3.2. Feed Fatty Acid Profile

Results obtained from fatty acid profile analysis of feed samples from the dietary treatments are presented in Table 3.

The fat content of the feed was rich in linolenic acid (~55%), followed by oleic acid (~33%), and a content in saturates approx. 12%, as expected, considering the ingredients in Table 1.

### 3.3. Feed Antioxidant Parameters

Oleuropein content (OLE), total phenol content (TPC), total flavonoid content (TFL), and in vitro antioxidant activity (DPPH^●^ scavenging and cupric ion reducing antioxidant capacity, CUPRAC) of the feeds are given in Table 4.

Oleuropein was present in the diets that contained olive leaf extract (T2, T3) in a dose-dependent manner (in T3, ~2.3-fold higher OLE level was found compared to T2), as verified by HPLC. Due to the presence of the extract, TPC, TFL, and the antioxidant potential of the feeds were elevated compared to T1 and the feed containing the encapsulated oregano oil (T4). In the latter, all values were comparable to those of the T1. T3, as expected, was the richest in bioactives and with a higher antioxidant potential.

### 3.4. Characterization of Essential Oil Content and Composition

The GC–MS analysis revealed that thymol and carvacrol were the main components of the essential oil. The major compounds identified are presented in Table 5. Antioxidant parameters evaluated in essential oil are also presented in Table 5.

### 3.5. Olive Leaf Extract Characterization

The parameters measured in olive leaf extract are presented in Table 6.

### 3.6. Egg Quality Parameters

For the overall experimental period, all treated eggs (T2, T3, T4) had improved eggshell weight and eggshell thickness than T1 (*p* < 0.001 for both parameters). The transverse axis was shorter in the T4 group (*p* = 0.022). Shape index was increased in T2 eggs (*p* = 0.029). T4 treatment affected eggshell color, which became lighter compared to the other treatments (*p* < 0.001). Haugh units were increased in T2 treatment (*p* < 0.001). Regarding egg yolk color, it was lighter in T3 yolks (*p* = 0.011), an effect which was also apparent by the lower a* values observed for the T3 treatment (*p* = 0.03). Yolk lightness (L*) was higher in the T2 group compared to the T4 group (*p* = 0.014). The results are presented in Table 7 for the 1st, 4th, and 6th week and the overall period. Overall period values were calculated, taking into account the measurements from all experimental weeks. As shown in Table 7, most egg quality parameters were not affected at the end of the 1st week, except for yolk color, which was lighter in the T2 and T3 groups in comparison with T1 (*p* = 0.031). At the end of the 4th week, the lightness (L*) of egg yolk was higher in T3 and T4 groups compared to T2 (*p* < 0.001). Moreover, the changes in eggshell weight and thickness became apparent in the 4th week’s measurements, with all treatments positively affecting eggshell weight and thickness compared to T1 (*p* = 0.004 and *p* = 0.012, respectively). At the end of the 6th week, all treatments continued to result in numerically higher eggshell weight and thickness compared to T1, but significant effects were noticed only for T2 treatment (*p* < 0.001 for both parameters), as shown in Table 7.

### 3.7. Egg Yolk Lipid Oxidation, Total Phenol Content (TPC) and Total Antioxidant Capacity (TAC)

In Day 1 of analysis, MDA levels in yolk were significantly lower in T2 and T4 groups compared to T1 and T3 (*p* < 0.001). On Day 5 of the analysis, there were no differences in yolk MDA levels among the treatments. Results are presented in Table 8. Egg yolk TPC expressed as μg of Gallic Acid equivalents per gram of dry yolk (μg GAE/g) was higher in T2 treatment compared to T3 and T4 (*p* = 0.026). Results are presented in Table 8. As shown in Table 8, Total Antioxidant capacity (TAC) was increased in T1 and T2 treatments in comparison with T4 (*p* = 0.003).

### 3.8. Egg Yolk Fatty Acid Profile

Egg yolk fatty acid analysis showed that the majority of the individual fatty acids were not affected by the treatments. However, T3 treatment increased the proportions of Margaric (17:0), Linoleic (C18:2n6c), α-Linolenic (C18:3n3) and cis,cis-11,14-Eicosadienoic (C20:2n6) fatty acids in egg yolk compared to T1 (*p* = 0.016; *p* = 0.010; *p* = 0.001; *p* = 0.020, respectively). The results are presented in Table 9.

**Figure 1:**
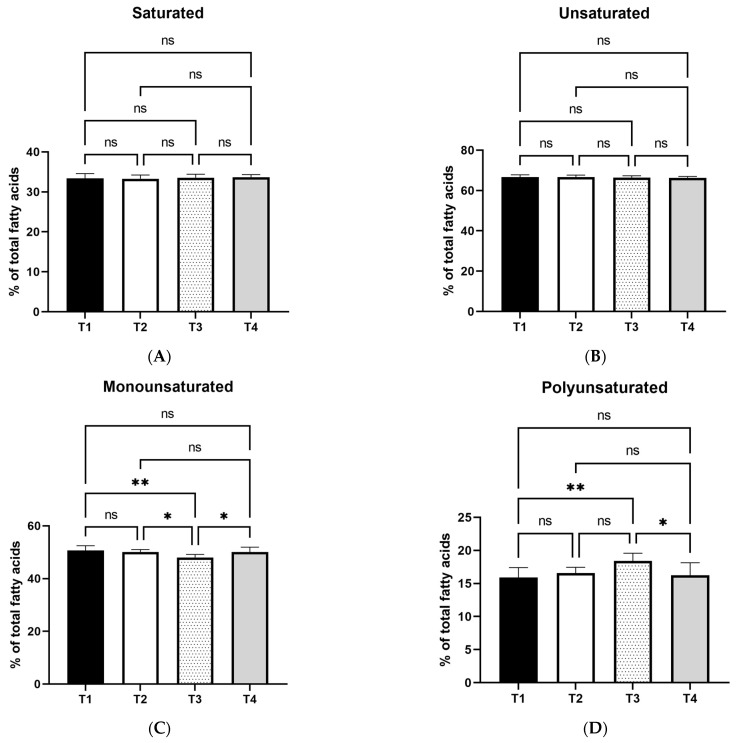
Effects of two levels of dietary olive leaf extract or oregano oil on egg yolk saturated (**A**), unsaturated (**B**), monounsaturated (**C**), and polyunsaturated (**D**) fatty acid percentage. T1: Control; T2: basal diet with 1% olive leaf extract; T3: basal diet with 2.5% olive leaf extract; T4: basal diet with 0.1% encapsulated oregano oil. *: mean values differ significantly between them (*p* < 0.05); **: mean values differ significantly between them (*p* < 0.01); ns: not significant. (*n* = 8).

**Figure 2:**
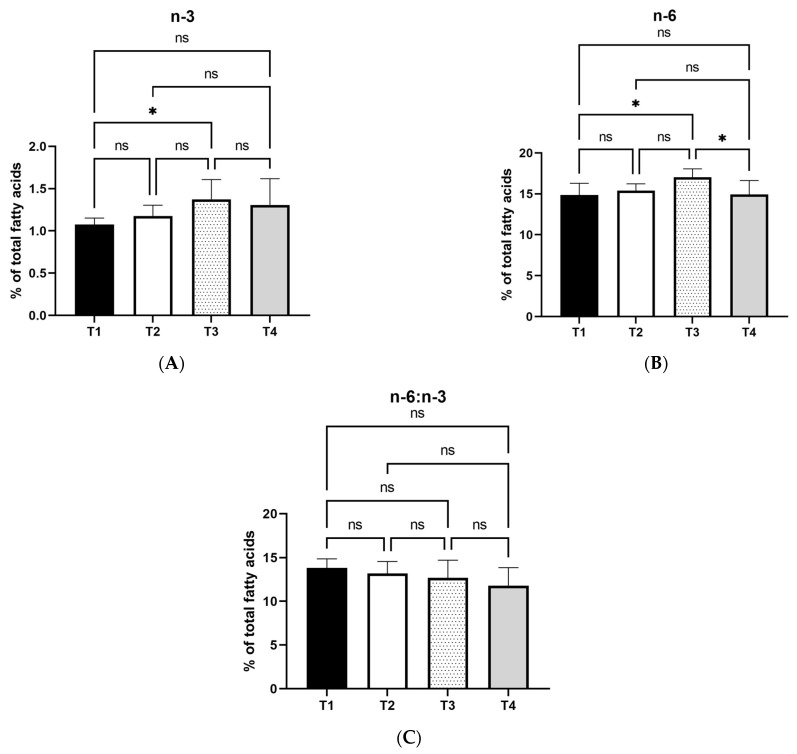
Effects of two levels of dietary olive leaf extract or oregano oil on n-3 (**A**), n-6 (**B**), and n-6:n-3 ratio (**C**) of egg yolks. T1: Control; T2: basal diet with 1% olive leaf extract; T3: basal diet with 2.5% olive leaf extract; T4: basal diet with 0.1% encapsulated oregano oil. *: mean values differ significantly between them (*p* < 0.05); ns: not significant. (*n* = 8).

Following the determination of major fatty acid categories, the statistical analysis results are presented in Figure 1 and Figure 2. No differences were detected in saturated and unsaturated fatty acid percentages among treatments. The monounsaturated fatty acid (MUFA) percentage was lower (*p* < 0.01) in the T3 group compared to T1. It was also lower compared to T2 and T4 (*p* < 0.05). The opposite effect was observed for T3 treatment on total polyunsaturated fatty acids (PUFA), which were found to be significantly higher compared to T1 (*p* < 0.01). Moreover, PUFA levels were lower in T4 treatment compared to T3 (*p* < 0.05).

In comparison with T1, T3 treatment resulted in higher n-3 and n-6 fatty acid content in egg yolks (*p* < 0.05). Moreover, according to statistical analysis, n-6 fatty acid levels were lower in T4 treatment compared to T3 (*p* < 0.05). The n-6:n-3 ratio was not affected by the treatments, as shown in Figure 2C.

### 3.9. Egg Yolk Cholesterol and α-Tocopherol Content

Yolk cholesterol content did not differ significantly among the groups (Table 10). Regarding α-tocopherol, all treatments had increased levels compared to T1 (Table 10). More precisely, according to statistical analysis, T3 yolks had significantly higher α-tocopherol content than T2 and T4, which in turn had significantly higher values than T1 (*p* < 0.001).

### 3.10. Pathology Evaluation

**Figure 3:**
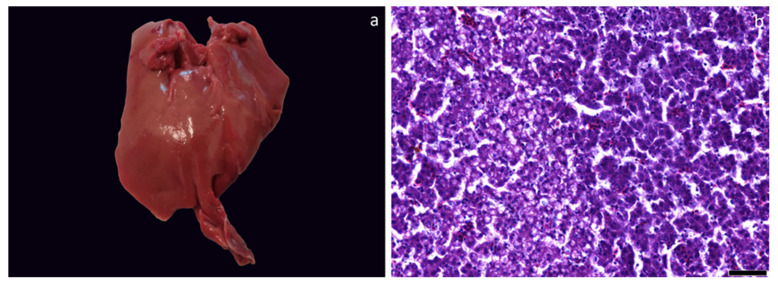
(**a**) Gross appearance of a mildly discolorated liver; (**b**) Focally extensive hepatocellular vacuolization (score 1). H&E, Bar = 50 μm.

Only liver discoloration was grossly observed, while histologically, multifocal areas of hepatic vacuolization were noted. The severity and the extent of the lesions ranged from mild to moderate, representing scores 1 and 2 on the four-tier system employed. However, among the groups, the lesions did not exhibit any distinct pattern in their distribution and did not show statistically significant differences (Figure 3, Table 11).

## 4. Discussion

As already mentioned, the olive supplement used in this study was a resin-purified aqueous isopropanol olive leaf extract, which was supplemented in laying hens in two levels. There was also a group supplemented with encapsulated oregano oil as a positive control. Oregano essential oil composition was generally in line with the literature for *Origanum vulgare* ssp. *hirtum* [42,43]. Given that the pure original oregano oil was unavailable for analysis, the values presented in Section 3.4 may be carefully evaluated. For instance, the absence of p-cymene and certain variations from the company’s declaration might be connected to the procedure of isolating the material from the encapsulated form [44].

Starting with the results of the treatments on egg quality, eggshell weight was increased in all treatments compared to T1, while some eggshell shape parameters (transverse axis, shape index) were also affected by the treatments. These changes in eggshell parameters may be attributed to the mineral content of olive leaves and oregano, especially calcium (Ca) and Phosphorus (P). It is widely known that Ca is an essential mineral for eggshell formation and quality, as eggshells consist mainly of CaCO_3_, with a percentage of about 94% [45]. It was found that Ca and P are among the major minerals found in olive leaves [46]. The latter authors also proposed olive leaf extract derived from three varieties as an affordable source of minerals such as Ca. In a previous study [47], two levels of olive oil were supplemented in laying hens, and the results showed increased eggshell-breaking strength and shell thickness. It was proposed by the latter authors that vitamin D, contained in olive oil, may be responsible for these effects; however, we cannot derive the same conclusion for our study, as the raw material used was different. In another study, olive pulp, a byproduct rich in minerals, resulted in a lower percentage of broken eggshells when supplemented in laying hens [4]. Possibly, a similar mechanism is involved in our study.

Oregano oil (T4) treatment resulted in lighter-colored eggshells, an effect that has not been previously reported, to our knowledge. Eggshell coloration is defined mostly genetically, but factors such as hen age, diseases, nutrition, stress, and environment can also affect it [48,49,50]. The main pigment responsible for the brown eggshell coloration is protoporphyrin IX, whereas biliverdin and its zinc chelates have little effect [51]. The last step in the protoporphyrin IX biosynthesis pathway is the auto-oxidation of protoporphyrinogen, a colorless molecule, to protoporphyrin IX [52]. In our case, possibly the antioxidants in oregano oil reduced this procedure, resulting in lighter-colored eggshells. On the other hand, no similar effects on eggshell coloration were noticed in the groups supplemented with olive leaf extract (T2, T3). The eggshells from these groups were slightly darker than those of T1. Even though olive leaf extract also contains high amounts of antioxidants, its different composition may be responsible for these inconsistencies. It has been reported that trace minerals such as iron (Fe) and magnesium (Mg) have positive effects with respect to brown eggshell coloration [50,53,54]. These minerals were found in high concentrations in olive leaves in the study of de Oliveira et al. (2023) [46], which provides a possible interpretation for our findings. Taking into account consumers’ preference for darker eggshells, maintaining the eggshell coloration in eggs from hens supplemented with 1% and 2.5% olive leaf extract is an important finding.

Haugh units are the most widely used measure of egg internal quality [55]. It is known that they deteriorate during storage time, and they also depend on hen age, but nutrition does not appear to have any great effect on albumen quality [55]. In some cases, diet modification can improve Haugh units, as shown for rosehip and flaxseed meal supplementation in laying hens [56]. In the present study, T2 treatment resulted in increased Haugh units, which is contrary to previous studies regarding olive byproduct supplementation in laying hens, where Haugh units were unaffected [4,57,58,59,60]. The different types and inclusion levels of byproducts used in these studies may be responsible for these inconsistencies. Moreover, in a previous study [61], 9% olive pulp supplementation in laying hens resulted in eggs with lower Haugh units compared to the control and lower supplementation level group (4.5%). In even higher inclusion levels of olive pulp, Haugh units were lower compared to control in the studies of Mohebbifar et al. [62] and Afsari et al. [63]. In the present study, the positive effect on Haugh units was noticed in the lower inclusion level, which is in accordance with the aforementioned studies. This finding might be due to the antioxidant compounds of olive leaf extract, which probably reduced albumen quality deterioration by reducing the lipid and protein oxidation procedures in the lower inclusion level.

Another important parameter that affects consumers’ preference is yolk coloration. Most European consumers prefer yolks with darker hues [64]. It is also known that yolk coloration depends on the accumulation of carotenoids in the diet, as carotenoids are synthesized de novo by some plant species, bacteria, algae, and fungi [65]. Laying hens cannot synthesize xanthophylls, which are the main carotenoids with pigmenting properties [65]. As olive leaves are considered an excellent source for the recovery of carotenoids [66], it was hypothesized that yolk coloration would be more intense in the groups supplemented with olive leaf extract. However, the results showed that the T2 treatment did not change yolk coloration compared to T1, while the higher inclusion level (T3) led to a lower yolk color fan score and redness value (a*). In a previous study [26], it was found that 2% and 3% olive leaf powder supplementation in laying hens improved egg yolk coloration. Literature about how dietary supplementation of olive leaf extract in laying hens affects egg quality is very limited. The carotenoid content of different olive leaf extracts may vary depending on the extraction method [67], which could be partially responsible for the unexpected finding of our study. Moreover, the lighter-colored yolks of the T3 group may indicate lower carotenoid content, which could be attributed to oxidation phenomena. Indeed, some bioactive compounds found in olive leaves, for example, phenolic compounds such as oleuropein, may exhibit pro-oxidant effects when supplemented at higher doses [68,69]. More plausible explanations may be either the reduction of the activity of the digestive enzymes by phenolic compounds when present at high levels in the diet [70,71], having as a consequence the limitation of the liberation of carotenoids from the feed matrix, or the reduction of micellization and the competition with carotenoids for introduction into the micelles [71]. The latter is a prerequisite before the absorption and transfer to target tissues. Regarding T4 treatment, oregano oil maintained yolk coloration. This finding is in line with a previous study [72], where oregano essential oil supplemented at two levels (50 or 100 mg/kg) in laying hens did not alter yolk coloration.

Lipid oxidation negatively affects animal product quality, organoleptic properties, nutritional value, and shelf-life [73]. MDA is a secondary lipid oxidation product, which is considered the main product for the evaluation of lipid peroxidation [74,75]. In the present study, both T2 and T4 treatments reduced yolk lipid oxidation in fresh eggs. In a previous study, dietary treatment with 10 g/kg olive leaves in laying hens resulted in lower yolk MDA values after 40 days of storage, but olive leaf extract supplementation led to values similar to control [25]. In another study, 10g/kg olive leaves supplementation in laying hens feeding with linseed oil-enriched diets did not alter MDA levels of yolks but reduced the concentration of lipid hyperoxides, which are primary lipid oxidation products [75]. It can be assumed that the olive leaf extract used in this study, which contained phenols and flavonoids responsible for its antioxidant properties, reduced lipid oxidation in the yolk. The same antioxidant molecules, however, could act as pro-oxidants in higher inclusion levels, which could explain the non-dose-dependent effect in the T3 group [68,69]. A similar interpretation was proposed in a previous study by our research group in broilers, where the same olive leaf extract was supplemented in two levels (1% and 2.5%), and the lower dose was more beneficial for the lipid oxidation of meat [5]. The effectiveness of dietary oregano essential oil in delaying lipid oxidation has been previously reported in egg yolks [76,77] and poultry meat [5,72,78,79,80].

Olive leaves contain a large variety of phenolic compounds with antioxidant properties, including simple phenols, flavonoids, and secoiridoids [81]. Literature about the deposition of total phenolic compounds in egg yolk is scarce. It has been shown that phenolic compounds found in poultry feed can be transferred and deposited in egg yolk [82,83], but the circumstances and the factors that are involved in phenolics’ bioavailability and deposition in egg yolk are still under investigation. In the present study, T4 feed samples had similar TPC to T1, but TPC was more than 2.5-fold higher in T2 diet and almost 5-fold higher in T3 diet in comparison with T1. Regarding egg yolk TPC, it did not differ significantly in any treatment in comparison with T1. This result may be attributed to the hydrophilic nature of the main phenolic compounds of olive leaves, i.e., oleuropein and hydroxytyrosol, which may limit their deposition in egg yolk [81,84]. However, the lower olive leaf extract inclusion level (T2 treatment) resulted in significantly higher TPC in yolk compared to the higher (T3). This finding indicates lower oxidation of phenolic compounds and may be related to the assumption that some molecules may act as antioxidants in low doses and pro-oxidants in higher doses. This mechanism has already been discussed previously, as this finding is in line with our previous results of T2 treatment in other antioxidant parameters (carotenoid content, MDA).

As emerging egg technology produces functional eggs by modifying the diets of laying hens, there is growing interest in preventing the oxidative deterioration of eggs [85]. In the present study, the yolk fatty acid profile was improved in the T3 treatment, which exhibited higher PUFA percentage and n-3 and n-6 content, with a corresponding reduction in MUFA percentage. The increased percentage of some individual PUFA (linoleic, α-linolenic, cis-cis-11,14 eicosadienoic) observed in the T3 treatment agrees with these findings. It is known that highly unsaturated eggs may be more prone to oxidation than conventional eggs [85]. Estimation of total antioxidant capacity (TAC) in yolk revealed that yolks from T2 and T3 groups, treated with olive leaf extract, maintained their antioxidant capacity, as it did not differ significantly from the one of T1. Despite the T3 group having the most unsaturated profile, the TAC values of yolk were reduced only numerically compared to T1 and T2. The lowest TAC values were those of T4 yolks, which were significantly lower compared to T1 and T2, and maybe this effect is due to differences in the content of antioxidants, pro-oxidants, and substrates prone to oxidation. In previous studies, 2% or 2.5% olive oil supplementation in laying hens increased unsaturated fatty acids (UFA) content in yolk [47] or PUFA content and the proportions of oleic and linolenic acids in yolk, respectively [60]. Dietary olive pulp also improved egg fatty acid profile in laying hens in the study of Dedousi et al. [4], with increased PUFA percentage and reduced SFA being the main findings. Regarding olive leaf extract’s effect on yolk fatty acid profile, the literature is very limited. Olive leaf extracts have been used successfully for stabilization purposes in refined olive oil, food lipids, and table olives [86,87,88]. Due to the presence of compounds with antioxidant properties in high concentrations, the olive leaf extract used in our study may have exhibited protective effects on yolk lipids, as shown by the higher PUFA percentage, n-3 and n-6 content, when supplemented in the higher dose. This observation warrants further investigation.

Yolk cholesterol levels were not affected by the treatments even though the limited data in the literature regarding the supplementation of olive oil, leaves (extract or powder), olive pomace, or even oleuropein indicated a beneficial effect. More specifically, in the study of Ahmed et al. [24], who added ~160–500-fold lower levels of the extract (50, 100, and 150 mg/kg) than in our study to the diet of Bandarah chicken, found a positive effect. The effect was more pronounced by increasing the levels of the extract. In another study [60], where two varieties of olive oil were supplemented in laying hens, one with high TPC and the other with low TPC, the results showed that only the high-TPC oil reduced plasma and yolk cholesterol levels compared to a control diet. Olive leaf powder up to 3% in laying hens’ diet tended to reduce yolk cholesterol [26]. Based on the existing literature, it can be assumed that the different olive products and byproducts, the different doses used, and the presence of oxidants, prooxidants, and nutrition/anti-nutrition factors in the diet may be responsible for the inconsistencies of the findings. More specifically, polyphenols, catechins, and flavonols have been associated with lowering cholesterol effects in egg yolk [89], while the type of dietary fat can also affect egg yolk cholesterol content [90]. Few studies have approached more precisely the possible underlying mechanisms, like the one of Iannaccone et al. (2019) [91], who found that dietary supplementation of dried olive pomace in laying hens modulated several biological pathways related to cholesterol biosynthesis. However, it should be mentioned that yolk cholesterol content is very resistant to changes, as embryo development normally requires a minimum necessary cholesterol concentration in yolk [92], so manipulating yolk cholesterol by hen nutrition is usually unsuccessful. As olive leaf extract is rich in phenolic compounds, further research is proposed to explore if there is potential in reducing yolk cholesterol levels and under which circumstances.

Olive leaves are considered an alternative α-tocopherol source, and appropriate extraction methods can lead to extracts with high α-tocopherol concentration [93]. Generally, yolk α-tocopherol content reflects the tocopherol concentration of the diet [94]. The findings of the present study were as expected, with α-tocopherol yolk enrichment being dose-dependent and, thus, higher in T3 treatment. They also agree with previous studies [67,85], where eggs of hens supplemented with olive leaves had higher α-tocopherol yolk content compared to the control group. Besides the apparent dose-dependent effect, it has been reported by many authors that the presence of other antioxidants in the diet could spare α-tocopherol and further increase its bioavailability by protecting it from oxidative damage during digestion in the intestine [75,94,95,96]. In a previous study, sage supplementation resulted in astonishingly higher yolk α-tocopherol content than expected [97], and the latter authors suggested that more tocopherol is available and absorbed by the intestine when other antioxidants are present in the diet of laying hens. The effect seen for oregano oil treatment (T4), which increased yolk α-tocopherol content compared to T1, could be related to the fact that the oil, though used at low levels, is encapsulated; thus, its phenols (mainly thymol and carvacrol), lipophilic in nature, are available in the intestine [98] favoring tocopherol protection. Taken together, it can be suggested that olive leaf extract supplementation in laying hens can increase α-tocopherol content in yolk not only due to its α-tocopherol content but also because it seems to protect and enhance the absorption of the α-tocopherol found in other feed ingredients. Enriching eggs with α-tocopherol could be important for consumer health, as α-tocopherol is the most bioactive form of vitamin E and has been associated with lowering the risk of chronic disease and protection against negative effects of aging and cognitive decline [99].

The liver was subjected to gross and histological analysis, which demonstrated that the incorporation of olive leaf extract had a beneficial effect. This inclusion did not have any negative impact on the lipid liver metabolism of the laying hens. Additionally, it resulted in the preservation of a heightened antioxidant status, as well as the promotion of enhanced fatty acid quality in egg yolks. Furthermore, the cholesterol levels remained stable throughout this process.

## 5. Conclusions

Overall, it can be concluded that a 1% level of dietary inclusion of a resin-purified aqueous isopropanol olive leaf extract in laying hens can improve albumen quality, reduce yolk lipid oxidation procedures, and increase yolk TPC. The greater supplementation level (2.5%) improved the yolk fatty acid profile but resulted in brighter yolk coloration. Oregano oil treatment, used as a positive control, reduced yolk MDA values of fresh eggs. All treatments increased eggshell weight and yolk α-tocopherol content, which was greater in the treatment with the higher dose of olive leaf extract (2.5%).

## Figures and Tables

**Table 1:** Ingredients and nutrients of the diet fed to laying hens during the experimental period.

Ingredients (%)	
Wheat soft	6.0
Corn	51.7
Soybean meal (47% crude protein content)	22.0
Wheat bran	7.5
Soybean oil	1.25
Limestone	9.65
Monocalcium phosphate	0.7
Sodium chloride	0.25
Sodium bicarbonate	0.24
DL-Methionine	0.19
Choline	0.08
Premix of vitamins and minerals *	0.44
Calculated analysis (%)	
Crude protein	17.0
Crude fiber	2.65
Crude fat	3.78
Crude ash	13.05
Metabolizable Energy (kcal/kg of diet)	2764

* Provided per kg diet: Retinyl acetate: 4.2 mg; Cholecalciferol: 0.1 mg; α-tocopherol acetate: 31.25 mg; Menadione: 5.0 mg; Cyanocobalamin: 0.025 mg; folic acid: 1.0 mg; Choline chloride: 450 mg; Pantothenic acid: 12.5 mg; Riboflavin, 6.25 mg; Nicotinic acid: 43.75 mg; Thiamin: 3.0 mg; D-biotin: 0.1 mg; Pyridoxine: 5.0 mg; Manganese: 125 mg; Zinc: 112 mg; Iron: 62 mg; Copper: 10 mg; Iodine: 1.0 mg; Selenium: 0.15 mg.

**Table 2:** Feed color, a_w_, and proximate composition.

Treatments					
Parameters	T1	T2	T3	T4	*p*-Value
L*	44.9 ± 0.2 ^a^	43.4 ± 0.1 ^b^	41.2 ± 0.3 ^c^	45.2 ± 0.3 ^a^	<0.001
a*	3.2 ± 0.2 ^a^	2.3 ± 0.1 ^b^	2.4 ± 0.3 ^b^	3.0 ± 0.1 ^a^	<0.001
b*	14.8 ± 0.1 ^a^	15.6 ± 0.2 ^b^	16.5 ± 0.5 ^c^	14.26 ± 0.1 ^a^	<0.001
ΔΕ	-	1.9± 0.2 ^a^	4.1 ± 0.3 ^b^	0.6 ± 0.2 ^c^	<0.001
Water activity (a_w_)	0.583 ± 0.012 ^a^	0.594 ± 0.004 ^a,b^	0.584 ± 0.002 ^a^	0.601 ± 0.004 ^b^	0.032
% Moisture	10.23 ± 0.18	10.24 ± 0.04	10.30 ± 0.51	10.25 ± 0.30	0.852
% Crude fat	2.7 ± 0.2	2.9 ± 0.2	2.7 ± 0.2	2.8 ± 0.1	0.384
% Crude protein	17.3 ± 0.1 ^a^	16.5 ± 0.6 ^a,b^	15.9 ± 0.3 ^b^	16.2 ± 0.4 ^b^	0.018
% Crude ash	13.0 ± 0.1 ^a^	14.0 ± 0.2 ^b^	13.5 ± 0.2 ^c^	11.6 ± 0.5 ^d^	<0.001

T1: Control; T2: basal diet with 1% olive leaf extract; T3: basal diet with 2.5% olive leaf extract; T4: basal diet with 0.1% encapsulated oregano oil. Values are means ± SD (*n* = 5 for L* to ΔΕ, *n* = 3 for all proximate composition values). Values in the same row with different superscripts differ significantly (*p* < 0.05).

**Table 3:** Fatty acid composition (%) of crude fat contained in feed samples.

Treatments					
Fatty Acid	T1	T2	T3	T4	*p*-Value
16:0	10.9 ± 0.2	10.5 ± 0.2	10.7 ± 0.1	10.9 ± 0.2	0.075
18:0	1.3 ± 0.2	1.3 ± 0.2	1.5 ± 0.1	1.6 ± 0.2	0.182
18:1 (n-9)	32.6 ± 0.7	33.9 ± 0.8	32.3 ± 0.7	32.8 ± 0.0	0.065
18:2	54.8 ± 0.4 ^a,b^	53.9 ± 0.3 ^b^	55.0 ± 0.6 ^a^	54.3 ± 0.3 ^a^	0.046
18:3 (n-6)	0.4 ± 0.0	0.6 ± 0.0	0.5 ± 0.1	0.5 ± 0.1	0.052

T1: Control; T2: basal diet with 1% olive leaf extract; T3: basal diet with 2.5% olive leaf extract; T4: basal diet with 0.1% encapsulated oregano oil. Values are means ± SD (*n* = 3). Values in the same row with different superscripts differ significantly (*p* < 0.05).

**Table 4:** Oleuropein content (OLE), total phenol content (TPC), total flavonoid content (TFL), DPPH scavenging (DPPH), and cupric ion reducing antioxidant capacity (CUPRAC) of feed samples.

Treatments					
Parameters	T1	T2	T3	T4	*p*-Value
OLE (mg/kg)	-	1852 ± 15 ^a^	4283 ± 27 ^b^	-	<0.001
TPC (mg GAE/g)	1.07 ± 0.04 ^a^	2.80 ± 0.05 ^b^	5.10 ± 0.06 ^c^	1.16 ± 0.04 ^d^	<0.001
TFL (μg QUE/g)	26.9 ± 1.5 ^a^	192.7 ± 2.2 ^b^	293.4 ± 1.9 ^c^	33.8 ± 1.3 ^d^	<0.001
DPPH^●^ (μmol TE/g)	4.7 ± 0.1 ^a^	14.1 ± 0.2 ^b^	27.8 ± 0.4 ^c^	5.2 ± 0.1 ^d^	<0.001
CUPRAC (μmol TE/g)	11.1 ± 1.2 ^a^	27.8 ± 2.2 ^b^	48.4 ± 1.0 ^c^	12.3 ± 1.3 ^a^	<0.001

T1: Control; T2: basal diet with 1% olive leaf extract; T3: basal diet with 2.5% olive leaf extract; T4: basal diet with 0.1% encapsulated oregano oil. Values are means ± SD (*n* = 3). Values in the same row with different superscripts differ significantly (*p* < 0.05).

**Table 5:** Essential oil content, composition, total phenol content (TPC), DPPH scavenging (DPPH), and cupric ion reducing antioxidant capacity (CUPRAC).

Essential Oil Major Compounds (%)	
Carvacrol	63.91
Thymol	24.9
Caryophyllene	2.75
Caryophyllene oxide	2.68
Bisabolene	2.68
Borneol	1.68
Terpineol	0.79
Trans dihydrocarvone	0.26
Essential Oil Antioxidant Parameters	
TPC (mg GAE/g oil)	60.2 ± 8.8
DPPH^●^ (mmol TE/g oil)	0.42 ± 0.03
CUPRAC (mmol TE/g oil)	0.68 ± 0.05

Values for antioxidant parameters are means ± SD (*n* = 3).

**Table 6:** Olive leaf extract water activity (a_w_), total phenol content (TPC), total flavonoid content (TFL), DPPH scavenging (DPPH), and cupric ion reducing antioxidant capacity (CUPRAC).

Olive Leaf Extract Characterization	
Water activity (a_w_)	0.238
TPC (mg GAE/g)	161.89 ± 7.0
TFL (μg QUE/g)	16.71 ± 0.07
DPPH^●^ (μmol TE/g)	1.05 ± 0.09
CUPRAC (μmol TE/g)	1.53 ± 0.13

Values for antioxidant parameters are means ± SD (*n* = 3).

**Table 7:** Egg quality parameters in the 1st, 4th, and 6th week of the experiment and for the overall experimental period.

Treatments					
Parameters	T1	T2	T3	T4	*p*-Value
1st Week					
Egg weight (g)	65.9 ± 4.92	64.7 ± 6.46	63.3 ± 5.59	62.6 ± 6.31	0.550
Yolk weight (g)	16.4 ± 0.60	16.3 ± 1.37	15.9 ± 1.20	16.4 ± 1.48	0.774
Albumen weight (g)	43.3 ± 4.29	42.2 ± 5.77	41.1 ± 4.09	39.8 ± 5.58	0.400
Eggshell weight (g)	6.19 ± 0.644	6.27 ± 0.859	6.25 ± 1.098	6.40 ± 0.714	0.949
Eggshell thickness (mm)	0.41 ± 0.040	0.40 ± 0.052	0.40 ± 0.053	0.41 ± 0.029	0.957
Longitudinal axis (mm)	59.5 ± 2.99	57.8 ± 1.89	58.6 ± 1.90	57.7 ± 1.92	0.183
Transverse axis (mm)	45.3 ± 1.25	45.4 ± 1.81	44.9 ± 1.09	44.8 ± 1.72	0.734
Shape index	76.3 ± 4.37	78.6 ± 2.76	76.6 ± 2.91	77.7 ± 2.65	0.315
Eggshell color	17.1 ± 2.40	18.6 ± 3.47	17.4 ± 3.45	19.4 ± 2.44	0.274
Yolk color fan score	12.6 ± 0.51 ^a^	11.8 ± 1.03 ^b^	11.8 ± 0.94 ^b^	12.1 ± 0.69 ^ab^	0.031
Haugh units	83.2 ± 8.68	88.1 ± 9.17	82.8 ± 7.12	85.2 ± 3.97	0.320
L*	74.7 ± 1.99	76.3 ± 2.18	74.7 ± 2.59	76.0 ± 1.11	0.133
a*	20.9 ± 1.77	19.3 ± 2.72	18.1 ± 3.03	18.8 ± 2.22	0.069
b*	55.1 ± 4.64	57.1 ± 4.54	53.4 ± 6.84	55.3 ± 5.30	0.438
4th Week					
Egg weight (g)	64.1 ± 5.52	66.0 ± 5.59	65.4 ± 3.70	63.3 ± 7.12	0.318
Yolk weight (g)	17.6 ± 1.75	17.4 ± 1.99	16.7 ± 1.51	17.4 ± 1.04	0.496
Albumen weight (g)	42.6 ± 5.12	41.7 ± 5.08	41.9 ± 3.25	41.4 ± 7.93	0.953
Eggshell weight (g)	5.52 ± 0.819 ^b^	6.65 ± 1.026 ^a^	6.43 ± 0.391 ^a^	6.30 ± 0.808 ^a^	0.004
Eggshell thickness (mm)	0.36 ± 0.043 ^b^	0.40 ± 0.048 ^a^	0.40 ± 0.018 ^a^	0.40 ± 0.036 ^a^	0.012
Longitudinal axis (mm)	58.9 ± 2.14	58.5 ± 2.20	58.9 ± 1.51	59.0 ± 3.00	0.954
Transverse axis (mm)	44.9 ± 1.46	45.5 ± 1.72	45.3 ± 1.18	44.8 ± 2.16	0.626
Shape index	76.2 ± 2.96	77.8 ± 2.80	76.9 ± 1.78	76.0 ± 2.54	0.238
Eggshell color	19.0 ± 3.56	17.9 ± 4.19	16.1 ± 1.91	19.0 ± 2.83	0.077
Yolk color fan score	11.4 ± 0.77	11.9 ± 0.73	11.5 ± 0.94	12.0 ± 0.82	0.147
Haugh units	75.6 ± 9.13	79.4 ± 9.96	75.3 ± 7.22	74.6 ± 6.13	0.425
L*	76.1 ± 3.12 ^ab^	74.0 ± 2.61 ^b^	78.5 ± 1.70 ^a^	77.6 ± 1.60 ^a^	<0.001
a*	17.1 ± 2.78	17.3 ± 2.29	16.3 ± 2.63	19.0 ± 2.62	0.075
b*	58.0 ± 6.21	56.8 ± 5.48	60.9 ± 4.07	60.7 ± 2.45	0.079
6th Week					
Egg weight (g)	64.7 ± 6.50	67.8 ± 5.26	63.2 ± 3.96	62.1 ± 8.14	0.116
Yolk weight (g)	17.0 ± 2.66	17.3 ± 1.77	15.9 ± 1.08	16.6 ± 1.86	0.271
Albumen weight (g)	42.2 ± 3.83	43.6 ± 4.73	41.2 ± 3.03	39.6 ± 6.91	0.211
Eggshell weight (g)	5.56 ± 0.935 ^b^	6.95 ± 0.933 ^a^	6.14 ± 0.538 ^b^	5.95 ± 0.657 ^b^	<0.001
Eggshell thickness (mm)	0.36 ± 0.047 ^c^	0.43 ± 0.052 ^a^	0.40 ± 0.019 ^b^	0.39 ± 0.027 ^b^	<0.001
Longitudinal axis (mm)	59.9 ± 2.71	59.7 ± 2.24	58.6 ± 1.61	58.0 ± 2.55	0.116
Transverse axis (mm)	44.9 ± 1.39	45.8 ± 1.14	44.8 ± 1.03	44.7 ± 2.12	0.224
Shape index	75.1 ± 2.89	76.8 ± 2.57	76.6 ± 2.28	77.1 ± 1.80	0.145
Eggshell color	17.9 ± 4.81	18.8 ± 3.20	16.9 ± 3.46	19.2 ± 2.90	0.398
Yolk color fan score	12.6 ± 0.65	12.7 ± 0.75	12.2 ± 0.90	12.3 ± 0.95	0.303
Haugh units	80.9 ± 11.71	83.3 ± 7.53	76.7 ± 8.34	78.2 ± 9.92	0.305
L*	75.1 ± 1.68	75.8 ± 2.65	75.5 ± 3.02	76.3 ± 1.83	0.603
a*	19.3 ± 3.14	19.5 ± 3.28	18.1 ± 2.94	18.3 ± 2.58	0.577
b*	54.8 ± 4.92	56.7 ± 6.31	55.3 ± 5.75	52.5 ± 6.09	0.327
Overall Period					
Egg weight (g)	65.2 ± 5.82	66.1 ± 5.82	65.4 ± 4.34	63.8 ± 7.89	0.066
Yolk weight (g)	17.2 ± 2.05	17.1 ± 1.74	16.6 ± 1.45	16.7 ± 1.44	0.089
Albumen weight (g)	42.7 ± 4.30	42.3 ± 4.95	42.3 ± 3.27	40.8 ± 7.00	0.124
Eggshell weight (g)	5.86 ± 0.859 ^b^	6.60 ± 0.952 ^a^	6.34 ± 0.761 ^a^	6.24 ± 0.816 ^a^	<0.001
Eggshell thickness (mm)	0.37 ± 0.042 ^b^	0.41 ± 0.048 ^a^	0.40 ± 0.037 ^a^	0.40 ± 0.036 ^a^	<0.001
Longitudinal axis (mm)	59.1 ± 3.01	58.6 ± 2.28	59.1 ± 1.84	58.6 ± 2.54	0.337
Transverse axis (mm)	45.1 ± 1.39 ^a^	45.6 ± 1.48 ^a^	45.2 ± 1.05 ^a^	44.8 ± 1.99 ^b^	0.022
Shape index	76.5 ± 4.56 ^b^	77.8 ± 2.68 ^a^	76.5 ± 2.38 ^b^	76.5 ± 2.38 ^b^	0.029
Eggshell color	18.1 ± 4.25 ^b^	17.9 ± 3.54 ^b^	16.9 ± 3.16 ^b^	20.4 ± 6.40 ^a^	<0.001
Yolk color fan score	12.1 ± 0.88 ^a^	12.1 ± 0.90 ^a^	11.8 ± 0.83 ^b^	12.1 ± 0.82 ^a^	0.011
Haugh units	79.6 ± 10.15 ^b^	83.9 ± 7.71 ^a^	77.7 ± 8.18 ^b^	78.3 ± 9.46 ^b^	<0.001
L*	74.9 ± 3.11 ^ab^	73.5 ± 4.09 ^a^	74.7 ± 3.79 ^ab^	75.4 ± 3.32 ^b^	0.014
a*	18.6 ± 3.00 ^a^	18.2 ± 2.85 ^a^	17.0 ± 2.65 ^b^	18.3 ± 2.72 ^a^	0.003
b*	55.0 ± 5.46	55.1 ± 7.13	55.8 ± 6.19	54.8 ± 6.23	0.779

T1: Control; T2: basal diet with 1% olive leaf extract; T3: basal diet with 2.5% olive leaf extract; T4: basal diet with 0.1% encapsulated oregano oil. Values are means ± SD (*n* = 12 for the 1st, 4th, and 6th week and *n* = 36 for the overall period). Values in the same row with different superscripts differ significantly (*p* < 0.05).

**Table 8:** Egg yolk MDA content evaluated on the 1st day after collection (Day 1) and after maintaining the samples in a refrigerator for 5 days (Day 5), total phenol content (TPC), and total antioxidant capacity (TAC).

Treatments					
Parameters	T1	T2	T3	T4	*p*-Value
MDA (ng MDA/g) Day 1	42.0 ± 17.42 ^a^	11.8 ± 10.41 ^b^	33.6 ± 12.70 ^a^	17.1 ± 8.26 ^b^	<0.001
MDA (ng MDA/g) Day 5	45.8 ± 28.38	25.3 ± 11.90	33.5 ± 13.95	33.7 ± 18.99	0.088
TPC (μg GAE/g dry yolk)	203.9 ± 74.64 ^ab^	230.0 ± 76.40 ^a^	171.2 ± 62.08 ^b^	189.5 ± 68.36 ^b^	0.026
TAC (%)	7.4 ± 2.15 ^a^	7.8 ± 3.52 ^a^	4.8 ± 6.06 ^ab^	1.8 ± 1.05 ^b^	0.003

T1: Control; T2: basal diet with 1% olive leaf extract; T3: basal diet with 2.5% olive leaf extract; T4: basal diet with 0.1% encapsulated oregano oil. Values are means ± SD (*n* = 12). Values in the same row with different superscripts differ significantly (*p <* 0.05).

**Table 9:** Egg yolk fatty acid profile (%).

		Treatments			
Fatty Acid	Τ1	Τ2	Τ3	Τ4	*p*-Value
C14:0	0.31 ± 0.043	0.32 ± 0.027	0.31 ± 0.027	0.34 ± 0.038	0.229
C14:1n5	0.10 ± 0.034	0.10 ± 0.026	0.08 ± 0.017	0.10 ± 0.032	0.618
C16:0	24.65 ± 1.320	24.43 ± 0.965	24.39 ± 1.060	24.99 ± 0.618	0.638
C16:1n9	0.70 ± 0.150	0.70 ± 0.096	0.69 ± 0.120	0.71 ± 0.104	0.972
C16:1n7	3.26 ± 0.723	3.23 ± 0.557	3.05 ± 0.569	3.41 ± 0.458	0.676
C17:0	0.17 ± 0.020 ^b^	0.18 ± 0.014 ^ab^	0.20 ± 0.019 ^a^	0.17 ± 0.018^b^	0.016
C17:1n7	0.11 ± 0.014	0.11 ± 0.018	0.12 ± 0.009	0.11 ± 0.020	0.382
C18:0	8.07 ± 0.616	8.17 ± 0.386	8.49 ± 0.508	8.01 ± 0.447	0.242
C18:1n9t	0.20 ± 0.453	0.18 ± 0.022	0.16 ± 0.010	0.19 ± 0.028	0.054
C18:1n9c	43.53 ± 2.206	42.98 ± 1.429	41.32 ± 1.521	42.74 ± 1.782	0.101
C18:1n7c	2.51 ± 0.133	2.57 ± 0.177	2.34 ± 0.166	2.55 ± 0.232	0.072
C18:2n6t	0.04 ± 0.017	0.04 ± 0.007	0.03 ± 0.005	0.04 ± 0.010	0.813
C18:2n6c	11.95 ± 1.438 ^b^	12.49 ± 0.866 ^ab^	13.96 ± 1.017 ^a^	11.93 ± 1.582 ^b^	0.010
C20:0	0.04 ± 0.012	0.04 ± 0.015	0.05 ± 0.026	0.04 ± 0.008	0.330
C18:3n6	0.09 ± 0.021	0.09 ± 0.018	0.07 ± 0.025	0.09 ± 0.013	0.202
C18:3n3	0.33 ± 0.048 ^b^	0.38 ± 0.044 ^ab^	0.47 ± 0.097 ^a^	0.34 ± 0.054 ^b^	0.001
C18:4n3	0.03 ± 0.016	0.04 ± 0.013	0.04 ± 0.005	0.04 ± 0.009	0.409
C20:1n9	0.28 ± 0.020	0.28 ± 0.025	0.27 ± 0.025	0.26 ± 0.016	0.662
C21:0	0.02 ± 0.005	0.02 ± 0.004	0.02 ± 0.003	0.02 ± 0.004	0.418
C20:2n6	0.13 ± 0.034 ^b^	0.15 ± 0.019 ^ab^	0.17 ± 0.023 ^a^	0.14 ± 0.030 ^ab^	0.020
C22:0	0.08 ± 0.021	0.08 ± 0.016	0.07 ± 0.010	0.07 ± 0.010	0.384
C20:3n6	0.13 ± 0.022	0.13 ± 0.011	0.14 ± 0.015	0.13 ± 0.021	0.812
C20:4n6	1.76 ± 0.125	1.70 ± 0.189	1.80 ± 0.154	1.72 ± 0.139	0.550
C23:0	0.05 ± 0.031	0.04 ± 0.011	0.03 ± 0.004	0.04 ± 0.015	0.260
C20:5n3	0.02 ± 0.006	0.02 ± 0.005	0.02 ± 0.003	0.02 ± 0.004	0.746
C22:4n6	0.18 ± 0.027	0.18 ± 0.030	0.20 ± 0.062	0.19 ± 0.038	0.706
C22:5n6	0.58 ± 0.155	0.61 ± 0.113	0.65 ± 0.168	0.69 ± 0.216	0.594
C22:5n3	0.10 ± 0.018	0.010 ± 0.023	0.11 ± 0.036	0.11 ± 0.025	0.464
C22:6n3	0.60 ± 0.048	0.63 ± 0.099	0.73 ± 0.194	0.79 ± 0.271	0.125

T1: Control; T2: basal diet with 1% olive leaf extract; T3: basal diet with 2.5% olive leaf extract; T4: basal diet with 0.1% encapsulated oregano oil. Values are means ± SD (*n* = 8). Values in the same row with different superscripts differ significantly (*p* < 0.05).

**Table 10:** Egg yolk cholesterol and α-tocopherol content.

Treatments					
Parameters	T1	T2	T3	T4	*p*-Value
Cholesterol (mg/100 g)	620.1 ± 186.5	613.6 ± 173.6	617.5 ± 156.6	547.1 ± 167.4	0.805
α-tocopherol (mg/100 g)	2.43 ± 0.5 ^c^	3.59 ± 0.9 ^b^	4.81 ± 0.9 ^a^	3.51 ±0.9 ^b^	<0.001

T1: Control; T2: basal diet with 1% olive leaf extract; T3: basal diet with 2.5% olive leaf extract; T4: basal diet with 0.1% encapsulated oregano oil. Values are means ± SD (*n* = 8). Values in the same row with different superscripts differ significantly (*p* < 0.05).

**Table 11:** The impact of the treatments on hepatic vacuolization scoring in laying hens is depicted as the percentage (%) distribution of cases within each treatment group across scoring categories. Score 1 indicates a mild presence of the lesion, while score 2 corresponds to a moderate presence of the lesion.

		Treatments			
Score	T1	T2	T3	T4	Asymptotic Significance (X^2^ Value)
1	100%	50%	83.3%	66.6%	0.217
2	0%	50%	16.7%	33.4%	

T1: Control; T2: basal diet with 1% olive leaf extract; T3: basal diet with 2.5% olive leaf extract; T4: basal diet with 0.1% encapsulated oregano oil (*n* = 6).

## Data Availability

Data are available upon request.
